# Maternal thyroid peroxidase antibody positivity and its association with incidence of low birth weight in infants

**DOI:** 10.3389/fendo.2023.1285504

**Published:** 2023-11-17

**Authors:** Liangmiao Chen, Dini Lin, Zhenzhen Lin, Enling Ye, Mengli Sun, Xuemian Lu

**Affiliations:** Department of Endocrinology, The Third Affiliated Hospital of Wenzhou Medical University, Ruian, Zhejiang, China

**Keywords:** pregnancy outcomes, thyroid peroxidase antibody, low birth weight infant, thyroid function, adverse pregnancy outcomes

## Abstract

**Background:**

Autoimmune thyroid disease is a prevalent condition affecting women of reproductive age, leading to thyroid dysfunction and impacting pregnancy outcomes. While the critical role of thyroid hormone in pregnancy outcomes is well-established, the potential association between positive anti-thyroid peroxidase antibodies (TPOAb) and adverse pregnancy outcomes in pregnant women with normal thyroid function remains unclear.

**Objective:**

This study aims to investigate the relationship between maternal TPOAb positivity and adverse pregnancy outcomes with normal thyroid function.

**Methods:**

We collected baseline information from pregnant women who visited our hospital between February 2009 and June 2012. Blood samples were taken to measure thyroid stimulating hormone (TSH), free thyroxine (FT4), TPOAb, and anti-thyroglobulin antibodies (TGAb). The incidence of adverse pregnancy outcomes was compared between TPOAb-positive and TPOAb-negative groups among participants with normal thyroid function.

**Results:**

A total of 7,046 pregnant women with normal thyroid function were included, comprising 6,700 with negative TPOAb and 346 with positive TPOAb. The TPOAb-positive group exhibited a higher age (26.0 vs. 27.0 years, *p* = 0.02) and greater serum TSH levels (1.72 vs. 1.94 mIU/L, *p* = 0.029), while the gestational week of blood collection was lower (31.9 vs. 26.5 weeks, *p* = 0.001). Univariate analysis revealed a higher incidence of low birth weight (LBW) in offspring of TPOAb-positive women compared to the TPOAb-negative group (3.5% vs. 1.9%, *p* = 0.035). After adjusting for confounding factors such as age, gestational week of blood collection, menstrual history, education level, gestational diabetes, gestational hypertension, TGAb, TSH, and FT4, TPOAb positivity emerged as an independent risk factor for LBW infants (OR: 2.317, 95% CI: 1.057–5.076, *p* = 0.036), while other adverse pregnancy outcomes did not show a significant correlation with TPOAb positivity.

**Conclusion:**

Our findings suggest that TPOAb-positive pregnant women with normal thyroid function are more likely to deliver LBW infants. Regular monitoring of TPOAb-positive pregnancies and timely interventions throughout all stages of pregnancy are crucial.

## Introduction

Autoimmune thyroid disease (AITD) is one of the most common diseases that affect women of reproductive age and result in thyroid dysfunction (1). In addition to thyroid dysfunction in pregnant women, it adversely affects pregnancy outcomes (2). Premature birth and newborn respiratory distress syndrome risk factors have been linked to maternal blood thyroid stimulating hormone (TSH) concentrations > 4 mIU/L in pregnancy by a roughly 2-fold increase (3). Additionally, a meta-analysis revealed that isolated hypothyroxinaemia is associated with a decreased risk of small for gestational age (SGA) and greater birth weight. However, subclinical hypothyroidism in pregnancy is linked to a higher risk of SGA and lower birth weight (4). Based on previous studies, the American Thyroid Association recommended that women with positive anti-thyroid peroxidase antibodies (TPOAb), TSH levels > 2.5 mU/L, and lower than the upper limit of the pregnancy-specific reference range consider levothyroxine replacement treatment (5). Overall, thyroid function is crucial in pregnancy outcomes and should be carefully considered.

In addition to thyroid hormones, the auto-antibodies generated during AITD, especially TPOAb, substantially impact pregnancy outcomes (6, 7). The prevalence of TPOAb positivity in pregnant women varies by region, race, and iodine consumption (8) and ranges from 5.0 to 18.9% (9, 10). However, it is still debatable whether positive TPOAb is associated with adverse pregnancy outcomes in pregnant women with normal thyroid function, such as premature birth and placental abruption (5, 11–13). Among 590 Japanese pregnant women, 10.9% tested positive for TPOAb (12). Levothyroxine usage could not lower the likelihood of high TPOAb titer, which was one of the risk factors for miscarriage. After conducting a prospective analysis of pregnant women between the ages of 18 and 40 with multiple pregnancies, it was (13) concluded that thyroid autoimmunity was not correlated with an elevated risk of premature birth, gestational diabetes, or preeclampsia. Therefore, the role of TPOAb in pregnancy outcomes still needs to be explored.

In this study, we conducted a retrospective analysis of 8,196 pregnancies to investigate the association between TPOAb positivity and adverse pregnancy outcomes.

## Materials and methods

### Participants

From February 2009 to June 2012, pregnant women who visited our hospital (the Third Affiliated Hospital of Wenzhou Medical University in Wenzhou City, Zhejiang Province, were recruited. The following categories were removed after testing their TSH and free thyroxine (FT4) levels: 1) Multiple pregnancies; 2) Stillbirth; 3) Symptoms of hypothyroidism or hyperthyroidism, as well as obvious signs like a goiter; 4) taking anti-thyroid or iodinated drugs (Levothyroxine: 25-150 μg per day; methimazole: 2.5-10 mg per day; propylthiouracil: 50-200 mg per day); 5) History of thyroid surgery or dysfunction; 6) Major diseases including heart failure, liver and renal insufficiency, malignant tumors, autoimmune diseases such as systemic lupus erythematosus, rheumatoid arthritis, and antiphospholipid syndrome; 7) Chronic diseases including diabetes and hypertension before pregnancy; 8) Pregnancy using assisted reproductive technology such as artificial insemination or vitro fertilization. The study’s design was approved by the Ethics Committee of the Third Affiliated Hospital of Wenzhou Medical University.

### Laboratory tests

The study collected retrospective data from pregnant women, including information on age, delivery history, education level, gestational week of blood collection, gestational week at delivery, gestational diabetes, gestational hypertension, birth weight, premature rupture of membranes, fetal distress, and Apgar score at 1 minute after birth. Venous blood samples were collected in the early morning while the subjects were fasting, and the serum was isolated and stored at -80°C for testing. The levels of TSH, FT4, TPOAb, and anti-thyroglobulin antibodies (TGAb) were measured by laboratory specialists.

TPOAb levels ≥ 50 IU/mL and TGAb levels ≥ 115 IU/mL were considered positive for determining antibody positivity. The blood samples were examined using the automated chemiluminescent immunoassay device DX2-800 with corresponding diagnostic reagents from Beckman, Germany. The intra-assay and inter-assay coefficients of variation for each serum index were below 10%.

### Adverse pregnancy outcomes

The recorded adverse pregnancy outcomes were as follows: LBW, premature rupture of membranes, premature birth, fetal distress, and low Apgar score in newborns at 1 minute after birth. Infants with LBW were defined as birth weight < 2500 g (14); premature rupture of membranes was defined as rupture of the amniotic sac and chorionic villi before labor (15); preterm birth was defined as delivery < 37 weeks of gestation; fetal distress was defined as fetal heart rate < 120 beats/min or > 160 beats/min, with meconium, fetal movement abnormalities, and fetal scalp pH < 7.2 (16); Apgar score < 7 was considered as low (17).

### Statistical analysis

Data processing and statistical analysis were performed using SPSS 23.0 statistical software (IBM Inc., USA). The skewed distribution data were presented as medians and quartiles and compared by the Mann-Whitney U test. The rank data were exhibited as numbers and percentages, and the comparisons were made by the chi-square analysis or Fisher’s exact test. In addition, the independent risk factors related to the adverse pregnancy outcomes were assessed by Logistic regression. *p* < 0.05 represented the statistically significant difference.

## Result

### Recruited participants and clinical characteristics

**Figure 1:**
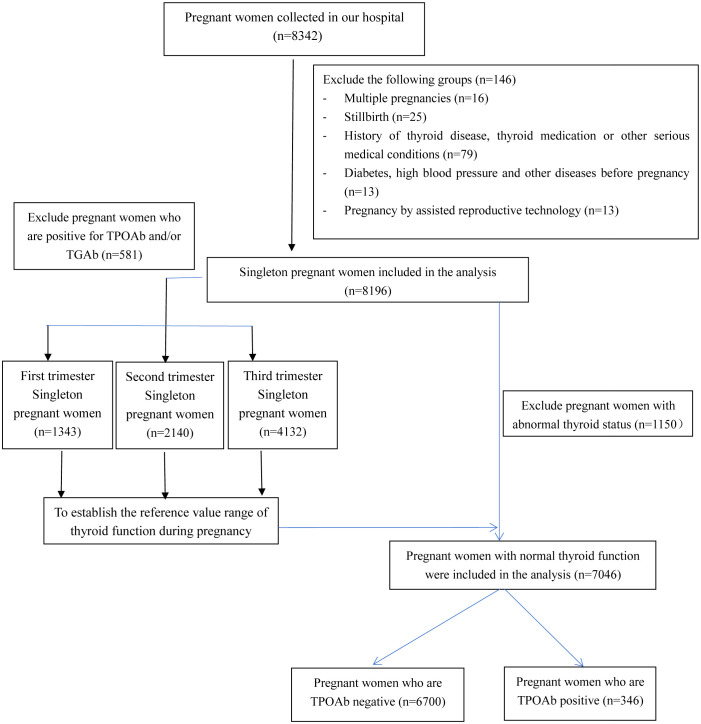
The flowchart for the inclusion of participants.

**Figure 1** illustrates the process of participant recruitment. Initially, 8,342 pregnant women were enrolled in the study. However, after excluding specific cases, such as 16 multiple pregnancies, 25 stillbirths, 79 pregnant women with hyperthyroidism or hypothyroidism symptoms or signs, individuals with known histories of thyroid disease or using thyroid-related medications, those with other significant medical conditions, 13 pregnancies involving assisted reproductive technology, 2 pregnancies involving diabetes, and 11 pregnancies with hypertension, a total of 8,196 pregnancies were included in the final analysis. Among these, 581 pregnant females tested positive for TPOAb and/or TGAb.

### Serum thyroid hormone reference range in recruited pregnancy females

TPOAb- and TGAb-negative pregnant females were used to define the reference value range of thyroid function in each pregnancy period. There were 1,343 instances in the first trimester (0-13 + 6 weeks), 2,140 cases in the second trimester (14-27 + 6 weeks), and 4,132 cases in the third trimester (≥ 28 weeks) (**Table 1**). Each trimester’s average age was 26 years old. The cut-off values of TSH and FT4 data were presented in percentiles due to the skewed pattern. For TSH, the median and 97.5% reference range values were 1.09 (0.14 to 3.62) mIU/L, 1.40 (0.45 to 3.35) mIU/L, and 2.20 (0.84 to 6.14) mIU/L in the first, second, and third trimesters of pregnancy, respectively (**Table 1**). In addition, the median and 95% reference range values for FT4 were 8.54 (6.63 to 11.25) ng/L, 6.73 (5.13 to 8.65) ng/L, and 6.77 (5.06 to 8.72) ng/L in the first, second and third trimesters of pregnancies (**Table 1**).

**Table 1:** The reference value range of thyroid function in each pregnancy period.

Pregnancy Trimester	Age (years)	TSH (mIU/L)			FT4 (ng/L)		
		2.5th	50th	97.5th	5th	50th	95th
First (n = 1343)	26 (24-28)	0.14	1.09	3.62	6.63	8.54	11.25
Second (n = 2140)	26 (25-29)	0.45	1.40	3.35	5.13	6.73	8.65
Third (n = 4132)	26 (24-30)	0.84	2.20	6.14	5.06	6.77	8.72

TSH, thyroid-stimulating hormone; FT4, free thyroxine.

### The differences between pregnant women with positive TPOAb and those with negative TPOAb

To examine the differences between pregnancies with positive TPOAb and those with negative TPOAb, 1,150 cases with abnormal thyroid function were excluded from 8,196 pregnancies. Among the 7,046 pregnant women with normal thyroid function, the incidence of positive TPOAb was 4.9% (346 cases) (**Figure 1**). There were significant differences in the age of pregnant women (26.0 vs. 27.0 years, *p =* 0.027) and the gestational week of blood collection (31.9 vs. 26.5 weeks, *p* = 0.001) compared with the TPOAb-positive group. In addition, the positive rate of TGAb and the serum TSH level was higher in the TPOAb-positive group (TGAb: 38.2% vs. 2.1%, *p* < 0.001; TSH: 1.94 vs. 1.72 mIU/L, *p* = 0.029). However, other variables, including gestational week at delivery, menopausal history, education level, incidence of gestational diabetes, incidence of hypertension during pregnancy, and FT4 levels, showed no significant differences between the two groups (all *p* values > 0.05) (**Table 2**).

**Table 2:** The baseline data of pregnant women with positive-TPOAb and those with negative-TPOAb.

Variables	TPOAb-negative (n = 6700)	TPOAb-positive (n = 346)	*P*
age (years), median (IQR)	26.0 (24.0-29.0)	27.0 (25.0-30.0)	0.027
gestational week of blood collection, median (IQR)	31.9 (17.6-38.7)	26.5 (13.7-38.4)	0.001
gestational week at delivery, median (IQR)	39.6(38.7-40.3)	39.6(38.7-40.3)	0.819
menopausal history, N (%)			0.472
No	5598 (83.6)	284 (82.1)	
Yes	1102 (16.4)	62 (17.9)	
education level, N (%)			0.122
Compulsory school	2816 (42.0)	160 (46.2)	
Upper secondary school	3884 (58.0)	186 (53.8)	
gestational diabetes, N (%)			0.673
No	6417 (95.8)	333 (96.2)	
Yes	283 (4.2)	13 (3.8)	
gestational hypertension, N (%)			0.441
No	6586 (98.3)	342 (98.8)	
Yes	114 (1.7)	4 (1.2)	
TGAb, N (%)			<0.001
No	6558 (97.9)	214 (61.8)	
Yes	142 (2.1)	132 (38.2)	
TSH (mIU/L), median (IQR)	1.72 (1.18-2.50)	1.94 (1.27-2.60)	0.029
FT4 (ng/L), median (IQR)	7.01 (6.27-7.83)	7.04 (6.28-7.82)	0.533

IQR, interquartile range; TPOAb, anti-thyroid peroxidase antibodies; TGAb, anti-thyroglobulin antibodies; TSH, thyroid-stimulating hormone; FT4, free thyroxine.

### The incidence of LBW increased in the TPOAb-positive group

In univariate analysis, the incidence of LBW among infants in the TPOAb-positive group was higher than that in the TPOAb-negative group (3.5% vs 1.9%, *p* = 0.035) (**Table 3**). However, there were no significant differences in the incidences of premature rupture of membranes, premature delivery, fetal distress, and low Apgar score at 1 minute after birth between the two groups.

**Table 3:** The differences in adverse pregnancy outcomes between pregnant women with positive TPOAb and those with negative TPOAb. .

Adverse pregnancy outcomes, N (%)	Total (n = 7046)	TPOAb-negative (n = 6700)	TPOAb-positive (n = 346)	*P*
LBW infants				0.035
No	6909 (98.1)	6575 (98.1)	334 (96.5)	
Yes	137 (1.9)	125 (1.9)	12 (3.5)	
premature rupture of membranes				0.540
No	6691 (95.0)	6360 (94.9)	331 (95.7)	
Yes	355 (5.0)	340 (5.1)	15 (4.3)	
premature delivery				0.878
No	6791 (96.4)	6457 (96.4)	334 (96.5)	
Yes	255 (3.6)	243 (3.6)	12 (3.5)	
fetal distress				0.217
No	6775 (96.2)	6438 (96.1)	337(97.4)	
Yes	271 (3.8)	262 (3.9)	9 (2.6)	
low Apgar score at 1 minute after birth				0.079
No	6999 (99.3)	6658 (99.4)	341 (98.6)	
Yes	747 (0.7)	42 (0.6)	5 (1.4)	

TPOAb, anti-thyroid peroxidase antibodies; LBW, low birth weight.

### TPOAb positivity was an independent risk factor for LBW

After adjusting for age, gestational week of blood collection, history of delivery, education level, incidence of gestational diabetes, incidence of gestational hypertension, TGAb, TSH, FT4 (infants with LBW incidence was also corrected for gestational week of delivery), the multivariate Logistic regression analysis found that TPOAb positivity was an independent risk factor for the incidence of LBW (OR: 2.317, 95% CI: 1.057–5.076, *p* = 0.036). However, premature rupture of membranes, premature delivery, fetal distress, and low Apgar score 1 min after birth showed no obvious correlation with TPOAb positivity (**Table 4**).

**Table 4:** The correlation between adverse pregnancy outcomes and maternal TPOAb positivity.

Adverse pregnancy outcomes	OR (95% CI)	*P*
LBW	2.317 (1.057–5.076)	0.036
premature rupture of membranes	0.929 (0.525–1.642)	0.799
premature delivery	0.904 (0.472–1.732)	0.762
fetal distress	0.702 (0.341–1.445)	0.337
low Apgar score at 1 minute after birth	1.821 (0.606–5.478)	0.286

CI, confidence interval; OR, odds ratio; LBW, low birth weight.

## Discussion

In this study, we conducted a novel retrospective analysis to explore the association between TPOAb positivity and adverse pregnancy outcomes in pregnancies with normal thyroid function. Our results revealed a significant increase in the incidence of LBW infants in the TPOAb-positive group. Moreover, we identified TPOAb positivity as an independent risk factor for LBW incidence. These findings emphasize the importance of close monitoring and promptly intervening in TPOAb-positive pregnant women throughout pregnancy.

In recent years, the routine screening of thyroid disorders in pregnant women has been a hot topic of debate in obstetrics and endocrinology. The cost-benefit ratio of early thyroid function and AITD screening for pregnant women was performed in the United States in 2008, which discovered that routine TSH and TPOAb tests prevented concerns about offspring and cost substantially more than routine testing. They once more indicated in 2012 that universal TPOAb screening for all pregnant women in the first trimester should be recommended, and that is much more cost-effective than neither no screening nor screening only for high-risk pregnant women (18). Furthermore, a cost-benefit analysis revealed that early universal thyroid screening is advantageous (19). Although early thyroid function testing, especially TPOAb testing, is essential, the underlying connection between TPOAb and adverse pregnancy outcomes is still not fully understood. Our study demonstrated that the infants in the TPOAb-positive group had a considerably greater incidence of LBW and that TPOAb positivity was an independent risk factor for LBW incidence.

LBW can cause the fetus to have a weak immune system after delivery, making it more susceptible to infection, and it can also cause the neural and linguistic systems to develop more slowly (20, 21). LBW and premature birth were more common in TPOAb-positive pregnant women, and LBW was linked to lighter premature infants (22). On the other hand, although TPOAb was not related to newborn weight, it was connected with premature rupture of membranes and postpartum thyroiditis (23) in China. TPOAb positivity, however, was revealed by our study to be a non-independent risk factor for premature birth but an independent risk factor for LBW. The fact that the kits used to detect TPOAb were all unique might have caused these inconsistent results. The findings of recent investigations contradict those of earlier research (24–27). More stricter test design and measurement standards, as well as a larger sample size, are needed to fully understand the impact of TPOAb on these pregnancy outcomes.

TPOAb may cause LBW through the following mechanisms: 1) TPOAb positivity indicates thyroid autoimmunity and pregnant women who test positive for this antibody may already be at risk of having a relative thyroid function deficit. Around 20% of women at birth had TSH levels higher than the normal reference range. A prospective study in Europe (28) found that in TPOAb-positive pregnant women with normal thyroid function who had not been treated with levothyroxine, TSH levels gradually increased from an average of 1.7 ± 0.4 mIU/mL to 3.5 ± 0.7 mIU/mL from 12 weeks of gestation to delivery. This suggested that TPOAb-positive patients’ residual thyroid function can still support pregnancy during the early stage, but decompensation may cause the sick thyroid gland to exhibit different degrees of hypothyroidism in the third trimester of pregnancy; 2) Increased autoimmunity has the potential to cause aberrant immunological regulation in the placenta, which would compromise the placenta’s ability to supply nutrients to the fetus (29, 30).

While our study has provided valuable insights into the relationship between TPOAb positivity and adverse pregnancy outcomes in pregnancies with normal thyroid function, it is essential to acknowledge the existing limitations. Firstly, the study population was derived from a single location, and the study design was retrospective, potentially introducing biases. To strengthen the validity of our findings, future research should encompass multi-center prospective studies involving diverse populations. Secondly, our study is limited by the lack of certain clinical information, such as pre-pregnancy weight, history of infertility and/or a diagnosis of polycystic ovary syndrome, which may impact pregnancy outcomes (31–33). In future investigations, collecting comprehensive data, including relevant variables, will help minimize potential biases and provide a more comprehensive understanding of the associations under study.

In conclusion, our study has demonstrated a higher incidence of LBW infants in the TPOAb-positive group. TPOAb positivity was identified as an independent risk factor for LBW incidence in pregnancies with normal thyroid function. These findings underscore the importance of close monitoring and timely interventions for TPOAb-positive pregnant women throughout pregnancy.

## Data availability statement

The raw data supporting the conclusions of this article will be made available by the authors, without undue reservation.

## Ethics statement

The study’s design was approved by the Ethics Committee of the Third Affiliated Hospital of Wenzhou Medical University. All participating patients provided signed written content. The studies were conducted in accordance with the local legislation and institutional requirements. Written informed consent for participation in this study was provided by the participants’ legal guardians/next of kin.

## Author contributions

LC: Methodology, Resources, Software, Validation, Writing – original draft. DL: Investigation, Project administration, Supervision, Visualization, Writing – original draft. ZL: Data curation, Project administration, Software, Visualization, Writing – review & editing. EY: Investigation, Resources, Supervision, Validation, Writing – original draft. MS: Methodology, Project administration, Software, Visualization, Writing – review & editing. XL: Conceptualization, Data curation, Methodology, Project administration, Resources, Writing – review & editing.
